# Food Game: A Gamified Interventional Study to Promote Healthy Eating, Lifestyle Behaviours, and Sustainability in Italian High School

**DOI:** 10.3390/nu18030482

**Published:** 2026-02-01

**Authors:** Chiara Stival, Silvano Gallus, Alessandra Lugo, Eugenio Santoro, Viviana Lisci, Maria Teresa Gussoni, Anna Odone, Benedetta Chiavegatti, the Food Game Group

**Affiliations:** 1 Department of Medical Epidemiology, Istituto di Ricerche Farmacologiche Mario Negri IRCCS, 20156 Milan, Italy; chiara.stival@marionegri.it (C.S.); alessandra.lugo@marionegri.it (A.L.); 2 Department of Clinical Oncology, Istituto di Ricerche Farmacologiche Mario Negri IRCCS, 20156 Milan, Italy; eugenio.santoro@marionegri.it; 3 Department of Prevention, ATS Città Metropolitana di Milano, 20122 Milan, Italy; vlisci@ats-milano.it (V.L.); mgussoni@ats-milano.it (M.T.G.); bchiavegatti@ats-milano.it (B.C.); 4 Department of Public Health, Experimental and Forensic Medicine, University of Pavia, 27100 Pavia, Italy; anna.odone@unipv.it; 5 Medical Direction, Fondazione IRCCS Policlinico San Matteo, 27100 Pavia, Italy

**Keywords:** school-based intervention, healthy diet, sustainability, Mediterranean diet

## Abstract

**Background/Objectives**: Adolescence represents a critical period for the formation of lifestyle habits that often persist into adulthood, significantly shaping long-term health outcomes and contributing to the development of non-communicable diseases. This study aims to assess the impact of Food Game, a secondary school-based programme, delivered throughout the academic year, to promote healthy eating, physical activity, and sustainability awareness among students. **Methods**: As part of the Food Game programme, 184 adolescents aged 14–16 years from the Milan area (Italy) completed two questionnaires, administered before and after the intervention (November 2024, April 2025), evaluating dietary habits, lifestyle behaviours, and attitudes toward sustainability. This uncontrolled intervention study assessed dietary changes using a composite score [0–14], with higher scores indicating healthier eating patterns. Pre–post intervention differences were analysed using paired *t*-tests for continuous variables and McNemar’s test for categorical variables. **Results**: After participation in Food Game, a significant improvement in mean dietary score from 7.6 to 8.2 (*p* < 0.001) occurred. Overall, 28.3% of the students worsened their score and 53.2% improved (≥1-point increase), including a significant improvement (≥2-point increase) in 29.4%. Fruit, vegetable, and fish intake increased, while consumption of meat, processed meat, and snacks decreased (*p* < 0.05). Waste recycling did not change (94.6%), and tap water non-significantly increased. No significant changes were observed in water intake, physical activity, screen time, or addictive behaviours. **Conclusions**: These findings support the potential of peer-led gamified interventions to promote healthier eating in youth. Future controlled studies are required to rigorously evaluate the Food Game programme’s effectiveness in relation to adolescents’ diet, lifestyle, and sustainability habits.

## 1. Introduction

Dietary and lifestyle habits established during adolescence are strong predictors of health behaviours in adulthood, with long-term implications for the prevention of chronic diseases and the promotion of overall well-being [1,2]. Even modest improvements in diet have been shown to significantly reduce the risk of non-communicable diseases such as cancer and coronary heart disease [3,4]. For example, a systematic review [3] observed significant reductions in risk of coronary heart disease, stroke, and total cancer all-cause mortality for each 200 g/day increment in intake of fruit, vegetables, and fruit and vegetables combined [3]. Another study [4] showed a significant inverse association with cardiovascular mortality for each additional serving a day of fruit and vegetables [4].

In particular, the adoption of a healthy dietary pattern, such as the Mediterranean diet, is associated with numerous health benefits, including a reduced risk of cardiovascular disease, obesity, and metabolic disorders [5,6]. This dietary model is characterised by a high intake of fruits, vegetables, legumes, whole grains, fish, and extra virgin olive oil and a low intake of red and processed meats and sugary products. It is also recognised as one of the most sustainable eating patterns from an environmental and cultural perspective [5,7]. In particular, the Italian Society of Human Nutrition (SINU) has proposed a new graphical representation of the traditional Mediterranean dietary model. This model not only emphasises plant-based foods, but it adds sustainability principles, prioritising local, seasonal, and minimally processed foods while discouraging food waste [8].

Schools represent a strategic setting for the promotion of healthy behaviours [9], as they provide access to a large and diverse population of children and adolescents, and they can play a crucial role in reducing health inequalities. In this context, peer-led interventions have gained increasing attention, as they leverage the influence of peer dynamics, particularly strong during adolescence, to promote positive behaviour change [10]. Evidence from international studies has shown that peer education can be effective in improving nutritional habits, physical activity, and other health-related behaviours among youth [10,11]. In recent years, gamification, defined as the use of game elements in non-game contexts, has also emerged as a promising strategy to increase engagement, motivation, and learning in health promotion programmes [12,13]. Despite growing interest in both peer education and gamification, few studies have investigated their combined use in school-based interventions targeting adolescent dietary and lifestyle behaviours, particularly when sustainability components are included. This represents a relevant gap in the literature, as integrated models may offer synergistic benefits by enhancing both motivation and social reinforcement mechanisms.

The Italian school context provides a unique opportunity to explore these dynamics, given the country’s cultural link to the Mediterranean diet and national efforts to incorporate sustainability into educational curricula. In addition, the feasibility of implementing structured, peer-led programmes within standardised school schedules further supports this context. The present project [14,15,16] aims to address this gap. The Food Game programme involves students guided by peer leaders (peer-led structure) and integrates game elements through a team-based competition (gamification). Through this hybrid model, Food Game is expected to lead to measurable improvements in adolescents’ dietary habits, lifestyle behaviours, and sustainable food choices. Specifically, the intervention aims to promote adherence to healthy dietary patterns, such as the Mediterranean diet, increase awareness of the environmental impact of food choices, and encourage sustainable consumption behaviours. This study therefore evaluates changes in these domains among secondary school students following their participation in the Food Game programme. In addition, the study also aims to assess whether baseline sociodemographic or behavioural characteristics are associated with dietary improvement or worsening after participation in the Food Game programme. This analysis is intended to provide a more comprehensive understanding of the factors influencing dietary changes.

## 2. Materials and Methods

### 2.1. Procedure

Food Game is a well-established, peer-education-based programme targeting high school students, designed to promote healthy eating habits, physical activity, and sustainable consumption within the ATS Milan area (Milan, Italy) [14,15,16]. The peer education component is ensured as students who participated in previous editions of Food Game act as peer mentors for new participants, guiding and motivating their schoolmates throughout the programme. Within this uncontrolled intervention study, as a first mandatory step, all participants are required to watch three educational videos on healthy eating, sustainable nutrition, and active living. This ensures consistent exposure to the core educational messages across the entire sample. Each class participates as a team and selects 5 out of 30 available health promotion activities, delivered over the course of the academic year. The duration of the project is seven months, with all participants starting and completing the programme simultaneously. Although students can choose among different tasks, their choices are guided by mandatory macro themes (healthy eating, promoting physical activity, and sustainable nutrition), ensuring that all team pathways remain comparable. Importantly, each team can follow a unique path, selecting tasks according to their interests and creativity, while still covering all macro themes (Table 1).

All activities are monitored and verified by ATS coordinators, either directly during school- or community-based tasks or through submitted reports and social media documentation. All activities within the Food Game programme are designed to be inclusive and adapted when necessary to ensure safe and equitable participation for every student. Students participate through structured, team-based challenges, with activities occurring both at school and at home and often involving family members. Each team earns points based on the completion and quality of the tasks, increasing friendly competition between classes and reinforcing engagement through the gamified structure of the programme. Examples of tasks include conducting a household food waste investigation, producing presentations promoting the Mediterranean diet, creating digital content on healthy habits for social media, cooking healthy traditional dishes together, or creating a mural at school on one of the programme’s macro themes. Its peer-led structure, integration of social-media-based tasks, and use of real-life gamified challenges effectively engage adolescents, leveraging peer motivation and opportunities for digital content creation while promoting practical skills, collaboration, and digital literacy. By engaging in hands-on, collaborative challenges, students apply concepts in real life, discuss them with peers, and receive immediate feedback, which strengthens understanding and facilitates lasting changes in dietary habits. During the first month of the game, videos addressing the programme’s themes are made available to all class groups. 

After viewing the videos, each class must complete a 40-item learning questionnaire. Only those classes that answer at least 35 questions correctly officially enter the competition and may then select the actions they will undertake as a group to promote healthy eating, physical activity, and sustainability.

During the 2024–2025 school year, the programme was implemented in 15 classes (teams), involving a total of 292 students aged 14 to 16 responding to the questionnaire in November 2024 prior to participating in the Food Game programme. Three classes involving 75 students decided to withdraw from the programme. Of the remaining 217 students, 184 (84.8%) also responded to the second questionnaire in April 2025 at the end of their involvement. Complete-case analysis was applied, as only data from participants with both pre- and post-intervention questionnaires were included.

The study was conducted in accordance with the Declaration of Helsinki. The parents of all of the participants provided written informed consent for study participation.

### 2.2. Outcome Measures

Detailed information on dietary habits was collected using a food frequency questionnaire, which assessed the monthly consumption of specific food groups: fruits, vegetables, whole grains (including whole wheat pasta and rice, barley, spelt, buckwheat, and quinoa), legumes, fish, meat and processed meats, snacks, and soft drinks. Additionally, breakfast habits, preferred types of fats (extra-virgin olive oil, other vegetable oils, butter, or margarine), and mean daily water intake (mL/day) were recorded.

To quantify adherence to a healthy diet in line with the Mediterranean diet, a custom scoring system was developed based on consumption of specific food items, water intake, and breakfast habits. Details on dietary scoring (ranging 0–14) are reported in Table 2.

### 2.3. Independent Variables

The questionnaire collected sociodemographic data, including sex and school grade (second or third), and type of school (professional school, technical school, or high school). Anthropometric measures were also self-reported by students to calculate BMI, in line with common practice in epidemiological questionnaires targeted to adolescents [17]. Students were also asked to report the number of days per week in which they engaged in at least 60 min of moderate to vigorous physical activity, in accordance with current international recommendations for this age group [18]. This question, widely used in epidemiological studies on adolescents’ health, was taken from the Health Behaviour in School-aged Children (HBSC) questionnaire [17].

Risky behaviours were evaluated by asking participants whether they had used specific substances (conventional cigarettes, novel tobacco and nicotine-containing products, including electronic cigarettes [e-cig] and heated tobacco products [HTPs], or alcohol) in the past 30 days. Based on their responses, students were classified as current users or non-users.

Screen-related recreational behaviours were assessed in terms of mean daily hours spent playing video games, watching TV or online videos, using social media, or navigating the web.

Additionally, sustainability-related attitudes and behaviours were evaluated. Participants were asked about their usual source of drinking water (tap water, plastic bottles, or glass bottles) and their recycling habits.

### 2.4. Statistical Analysis

Continuous variables before and after the Food Game programme were compared using paired *t*-tests, while changes in categorical variables were assessed using McNamar’s test for paired proportions. Improvement and worsening in each food category, as well as in the overall dietary score, were calculated according to the scoring system presented in Table 1 by comparing the questionnaires administered before and after the Food Game intervention.

Multilevel random-intercept logistic regression models were fitted to account for the hierarchical structure of the data, with students nested within teams (classes). Teams were specified as a random intercept to capture between-class variability, whereas sex, age group, and type of school were included as fixed effects based on theoretical relevance. Two main outcomes were assessed: (i) a substantial improvement in dietary habits (change in dietary score ≥ 2) vs. no substantial improvement in dietary score (change in score < 2); and (ii) a worsening in dietary habits (change in dietary score < 0) vs. non-worsening in dietary score (change in score ≥ 0) after participation in the Food Game programme.

All analyses were performed using SAS 9.4 (Cary, NC, USA).

## 3. Results

Among the 184 adolescents who completed both questionnaires, participation in the Food Game programme was associated with a significant overall improvement in dietary habits. The mean dietary score increased from 7.6 (standard deviation, SD = 2.5) at baseline to 8.2 (SD = 2.5) at follow-up (*p* < 0.001), and over half of the students (53.2%) showed an improvement (≥1 point increase) in their individual score (Table 3). In line with the intervention objectives, students reported a higher frequency of fruit (*p* = 0.014), vegetable (*p* = 0.002), and fish consumption (*p* = 0.013) after the programme, while intake of meat (*p* = 0.004), processed meat (*p* = 0.004), and snacks (*p* = 0.007) decreased significantly. There were no significant changes in the type of preferred fat, with the majority of participants continuing to use extra-virgin olive oil (EVO) both before (86.4%) and after (84.8%) the intervention. Similarly, mean daily water consumption did not significantly increase over the study period.

In terms of sustainable behaviours, nearly all participants reported consistently practicing waste recycling in both periods (94.6%). Although not statistically significant, a slight increase was observed in the proportion of adolescents reporting the use of tap water, from 28.8% to 33.2% (Table 4). No significant changes were observed in physical activity levels or screen-related recreational behaviours following participation in the Food Game. A slight, non-significant increase in addictive behaviours was reported.

Overall, 29.4% of students achieved a substantial improvement in dietary score (≥2-point increase; Table 5). According to the multivariable multilevel models, females and younger participants were more likely to show improvements in dietary score, although these associations were not statistically significant (odds ratio, OR = 1.20; 95% confidence interval, CI: 0.62–2.31 for females compared to males; OR = 0.76; 95% CI: 0.37–1.55 for older participants compared to younger ones). A higher BMI at baseline was significantly associated with improved dietary scores (*p* for trend = 0.034). Adolescents engaging in addictive behaviours were more likely to show a worsening of dietary score, although the associations did not reach statistical significance (OR = 1.38; 95% CI: 0.51–3.74 for current smoking; OR = 1.75; 95% CI: 0.75–4.13 for e-cig or HTP; OR = 1.91; 95% CI: 0.98–3.72 for alcohol use).

## 4. Discussion

Following participation in the Food Game programme, over half of the participants reported some improvement, with 29% showing a substantial increase, while 28% experienced a worsening. The mean intake of fruits, vegetables, and fish increased, whereas consumption of meat, processed foods, and snacks declined. Waste recycling remained consistently high (95%), and the use of tap water showed a slight, non-significant increase. No significant changes were observed in physical activity, screen time, or addictive behaviours. Although the absence of a comparison group limits causal inference, the observed dietary improvements may represent foundational shifts with the potential to consolidate into sustained healthy habits.

The study found that the gamification-based Food Game programme is promising for promoting adolescents’ healthy behaviours, consistent with existing literature on this age group [13,19,20]. In particular, participants reported an increased intake of healthy foods, such as fruits, vegetables, and fish, and a reduction in the consumption of less healthy items, including red and processed meats and snacks. These improvements, even if modest, may represent early behavioural shifts with the potential to consolidate into long-term dietary habits, which are known to influence the risk of obesity and cardiometabolic diseases later in life. These results are in line with findings from international studies [20]. A recent systematic review of gamified interventions reported increased fruit and vegetable intake among adolescents following short-term programmes worldwide [20]. Another web-based, team-oriented intervention conducted in Canada resulted in a significant increase of fruit and vegetable servings per day among high school students participating in the programme, compared with controls [21], highlighting the potential of digitally-augmented and group-based interventions. Similarly, a cluster-randomised controlled trial implemented in Chile demonstrated that a school-based gamification strategy of healthy challenges led to reductions in BMI z-score among students in the intervention arm compared to control schools [22].

Our findings are particularly relevant in the Italian context, where a gradual shift away from the traditional Mediterranean diet has been observed [23], especially among younger generations [24]. It is noteworthy that participants showed relatively positive attitudes toward sustainability, indicating heightened awareness and sensitivity among young people regarding environmental issues. Given that the Mediterranean diet is also recognised as one of the most sustainable dietary models [5,7], this may have further motivated adolescents to align with it. While the programme shows potential in supporting healthier behaviours, it is important to note that a proportion of participants did not improve. Possible reasons for the lack of improvement include lower adherence to the intervention and individual or contextual factors (e.g., motivation, habits, sociodemographic characteristics) that may have limited participants’ engagement. However, these potential explanations were not directly investigated in the present study.

Screen-related habits remain a critical issue among youth, and the study did not observe any significant changes in these behaviours. This finding underscores the importance of ongoing monitoring, as screen time is closely linked to both physical and mental health outcomes [25]. Moreover, social media use is associated with lower fruit and vegetable intake and with an improvement of unhealthy snacks while scrolling [26].

A slight, although not statistically significant, increase in addiction-related behaviours was observed over the study period. This trend aligns with existing literature [27], which indicates that such increases are physiologically and psychologically common during adolescence. This developmental phase is marked by exploration and experimentation and is often accompanied by a heightened interest in potentially risky behaviours over relatively short time frames. According to data from one among the main European surveillance system on adolescents [28], a much sharper increase in prevalence would typically be expected in this age group. To determine whether the smaller increase observed in our study may be attributable to the Food Game intervention, a controlled study design would be necessary.

The study presents several strengths and limitations. Among its strengths are the innovative nature of the intervention, the high level of student participation. In addition, a comprehensive range of variables was included in the questionnaire and consistently collected at both time points, enabling an objective pre–post comparison. However, key limitations are the relatively to the absence of a control group, which prevents definitive conclusions about the causal impact of the Food Game programme on the observed changes. In addition, the lack of significant associations in the multilevel analysis may be due to the small sample size, indicating that larger studies are needed to better examine these associations. The lack of significant associations in the multilevel analysis may be due to the small sample size, suggesting that larger studies are needed to better examine these associations. Nonetheless, this result may also suggest that the Food Game programme produces similar effects across different sub-groups of adolescents, supporting its potential applicability in diverse school settings. Moreover, the drop-out rate was not negligible, which may have introduced additional uncertainty in the interpretation of the findings. Nevertheless, reporting preliminary results from the 2024–2025 school year provides a necessary starting point to inform and guide future research, including the planned implementation of a controlled study to rigorously assess programme efficacy. In addition, the dietary score is not validated, although 10 questions are common to those used for the KIDMED score (based on 16 items), one of the most frequently used validated dietary score for children and adolescents [29]. Moreover, despite food frequency questionnaires are commonly used to assess dietary habits in adolescent populations [30], the self-reported nature of the questionnaire represents a further limitation, as it may introduce recall or reporting bias. A key limitation of this study is the lack of precise age data for participants, as only age groups were available; this prevented the calculation of age- and sex-specific BMI cut-offs. Future studies should collect exact age of participants to be able to calculate age-specific BMI cut offs. Another limitation of the present study is the lack of information on students’ family socioeconomic or educational status, history of poor dietary habits and school characteristics. Consequently, the analysis was unable to assess whether students from more advantaged schools or areas performed differently from those in less advantaged contexts. Future research should collect such information to evaluate potential biases and ensure that gamified interventions are effective and equitable across diverse populations. The study also did not collect information on participants’ academic performance, which prevented us from exploring potential associations between academic performance and responsiveness to the intervention. Future studies should include such measures to better understand their influence on engagement and outcomes. In addition, specific behaviours, such as smoking status, are described in very general terms. Nevertheless, the sample size did not allow for more detailed classifications. A further limitation is that a complete-case analysis was performed, and we were unable to compare students who completed both questionnaires with those who withdrew or provided only baseline data. Therefore, potential differences between completers and non-completers could not be assessed. Moreover, future evaluations of the Food Game programme could benefit from incorporating objective measures, such as accelerometry for physical activity or detailed dietary records. In addition, extending follow-up periods would allow assessment of the maintenance of behavioural changes over time. These methodological improvements would provide more robust evidence on the effectiveness and sustainability of the Food Game intervention in promoting healthier lifestyle habits among adolescents. Moreover, given the large number of outcomes assessed, the possibility of type I error due to multiple comparisons cannot be excluded, especially in this exploratory study. No formal correction was applied, and this limitation should be considered when interpreting the results.

## 5. Conclusions

Overall, the implications of our findings suggest that school-based, peer education programmes integrating gamification, such as the Food Game, may support improvements in adolescent dietary habits. Participants reported increases in the consumption of fruits, vegetables, and fish and reductions in less healthy foods, indicating promising trends for healthier eating patterns. However, in the absence of a control group, these changes cannot be definitively attributed to the Food Game programme. These results highlight practical implications for schools and public health programmes, suggesting that integrating peer-led, gamified interventions into the school curriculum may enhance engagement and awareness of healthy and sustainable dietary practices. Future research should implement controlled, longitudinal designs to rigorously evaluate the effectiveness and long-term impact of the Food Game programme on adolescent dietary behaviours, lifestyle choices, and sustainable habits.

## Figures and Tables

**Table 1:** Description of the steps to be fulfilled by teams.

The intervention was structured around three predefined thematic areas: healthy eating, active lifestyle choices, and sustainable and environmentally friendly behaviours. Within the game, each class group had to fulfil 5 steps, 2 mandatory and 3 selected from a predefined list of steps. Besides the two mandatory activities (step 1 and step 30), each group was required to select and complete (i) one activity promoting healthy eating, (ii) one activity promoting active lifestyle choices, and (iii) one activity promoting sustainable and environmentally friendly behaviours. The full list of activities, from which each group selected and defined its own intervention pathway, is reported below.	
**The following two activities were mandatory for all participating groups:** **Team test (step 1):** assessment of knowledge related to the educational materials made available to all classes (3 educational videos). **Dissemination activity (step 30):** presentation of the Food Game programme and the progress achieved during the intervention period to students within the same school. **Steps specific to healthy eating** Creation of a logo for a healthy snack. Organisation of a fruit and vegetable day at school. Collective preparation of traditional healthy dishes. Development of a presentation promoting the Mediterranean diet. Critical analysis of a food advertisement, including identification of marketing techniques. **Steps specific to active lifestyle (physical activity)** Organisation of a multigenerational walk. Organisation of group walking initiatives for travelling to school. **Steps specific to sustainable and environmentally friendly behaviours** Conducting a family survey on household food waste. Participation in the “Food Game Ghostbusters” activity focused on plastic waste collection. Planning and execution of family grocery shopping for four days, including development of a healthy, sustainable, and balanced menu. Assessment of household packaging waste and production of a video with strategies to reduce it. Calculation of individual and family ecological footprints, followed by development of a team agreement on behaviours to improve. Monitoring of household waste for four days, including plastic, metal, paper, and cardboard, and joint development of a waste reduction handbook.	**Steps to be adapted to one of the three Food Game thematic areas** Some activities allowed class groups to promote one or more of the three thematic areas. These included Production and dissemination of awareness-raising materials. Organisation of a flash mob focused on one of the intervention’s thematic areas. Creation of a school mural addressing one of the thematic areas. Organisation and dissemination of a mail-based awareness campaign. Organisation of a debate between different teams on one of the thematic areas. Promoting change in school on one of the game’s themes Organisation of the cleaning of a green space and monitoring of its maintenance over the subsequent two weeks. Establishment of a vegetable garden at school, including observation of plant growth throughout the intervention period. Organisation of a supermarket-based “packaging awareness” activity to reflect on unnecessary packaging and its rapid disposal. Development of an original game-based activity addressing one of the Food Game’s macro themes. Teacher for a Day: organisation of a meeting with elementary school children and delivery of educational games addressing one or more thematic areas. Conducting a school-based survey on the initiatives implemented. Interviews with students who had participated in the Food Game during the previous year to explore self-reported behavioural changes. Organisation of a healthy food event incorporating movement-based games and recycling activities, accompanied by the development of promotional slogans. Environmental tutoring activities conducted in grocery stores, during which students encouraged customers to read nutrition labels and reflect on food packaging. This step was not applicable to the active lifestyle thematic area.

**Table 2:** Items of the food frequency questionnaire and dietary habits scoring system [score: 0–14].

Dietary Score Item	Points +0	Points +1	Points +2
Fats/oils	Butter; Other vegetal oils; Margarine	Extra-Virgin Oil	
Fruit	Never; Rarely (Sometimes a month); Once a week;	Sometimes a week	Once a day every day; More than once a day every day
Vegetables	Never; Rarely (Sometimes a month); Once a week;	Sometimes a week	Once a day every day; More than once a day every day
Whole pasta and rice and/or barley, spelt, buckwheat, quinoa	Never; Rarely (Sometimes a month); Once a week; Sometimes a week	Once a day every day; More than once a day every day	
Legumes	Never; Rarely (Sometimes a month); Once a week	Sometimes a week; Once a day every day; More than once a day every day	
Fish	Never; Rarely (Sometimes a month); Once a week	Sometimes a week; Once a day every day; More than once a day every day	
Meat (excluding processed meat such as cold cuts)	Sometimes a week; Once a day every day; More than once a day every day	Never; Rarely (Sometimes a month); Once a week	
Processed meat (such as cold cuts, salami, sausages, etc.)	Sometimes a week; Once a day every day; More than once a day every day	Never; Rarely (Sometimes a month); Once a week	
Snacks like chips, pastries, candies	Sometimes a week; Once a day every day; More than once a day every day	Never; Rarely (Sometimes a month); Once a week	
Soft drinks	Sometimes a week; Once a day every day; More than once a day every day	Never; Rarely (Sometimes a month); Once a week	
Glasses of water drunk/day	Less than 3 glasses a day; Between 3 and 7 glasses a day	At least 8 glasses a day (more than one litre)	
Breakfast	Never; Rarely (Sometimes a month); Once a week; Sometimes a week	Every day	
**Total**	**0–14**		

**Table 3:** Mean intake and self-rated changes (%) of major food groups and diet-related behaviours among 184 adolescents, before (November 2024) and after (April 2025) participation in the Food Game programme.

Type of Food Item	Intake/Month (Mean ± SD)		Changes in Intake After Food Game Programme N (%)		
	November 2024	April 2025	Worsening	Not Changing	Improving
*Food item*					
Fruit (↑)	24.2 ± 19.7 ^a^	27.4 ± 19.9 ^a^	34 (18.5)	90 (48.9)	60 (32.6)
Vegetables (↑)	26.7 ± 18.6 ^a^	30.3 ± 20.0 ^a^	23 (12.5)	104 (56.5)	57 (31.0)
Grains (↑)	25.4 ± 18.2	24.9 ± 16.8	47 (25.6)	86 (46.7)	51 (27.7)
Legumes (↑)	7.9 ± 7.4	9.3 ± 9.3	34 (18.5)	98 (53.2)	52 (28.3)
Fish (↑)	5.8 ± 5.1 ^a^	6.9 ± 5.1 ^a^	25 (13.6)	100 (54.3)	59 (32.1)
Breakfast (↑)	19.0 ± 12.5	19.9 ± 12.2	21 (11.4)	129 (70.1)	34 (18.5)
Meat (↓)	14.4 ± 11.3 ^a^	12.2 ± 8.4 ^a^	32 (17.4)	107 (58.1)	45 (24.5)
Processed meat (↓)	10.1 ± 8.7 ^a^	8.4 ± 7.2 ^a^	27 (14.7)	104 (56.5)	53 (28.8)
Snacks (↓)	19.1 ± 16.4 ^a^	16.0 ± 15.1 ^a^	41 (22.3)	68 (37.0)	75 (40.7)
Soft drinks (↓)	6.4 ± 10.2	6.9 ± 12.1	38 (20.7)	106 (57.6)	40 (21.7)
EVO as preferred fat (%) (↑)	86.4	84.8	15 (8.2)	157 (85.3)	12 (6.5)
Water consumption (ml/day) (↑)	905.2 ± 286.9	927.2 ± 291.4	29 (15.8)	119 (64.6)	36 (19.6)
Dietary score [0–14] (↑)	7.6 ± 2.5 ^a^	8.2 ± 2.5 ^a^	52 (28.3)	34 (18.5)	98 (53.2)

EVO: extra-virgin olive oil; SD: standard deviation. (↑) Items recommended for increased consumption: worsening = change in score < 0; improving = change in score ≥ 1. (↓) Items recommended for reduced consumption: worsening = change in score > 0; improving = change in score < 0. ^a^ *p* value of paired *t* test < 0.05.

**Table 4:** Characteristics and self-rated changes (%) among 184 adolescents before (November 2024) and after (April 2025) participation in the Food Game programme.

Characteristics	November 2024	April 2025	Changes After Food Game Programme N (%)		
			Worsening	Not Changing	Improving
Age (years), M ± SD	15.2 ± 0.5 ^a^	15.6 ± 0.5 ^a^	-	-	-
BMI ^b^, M ± SD	20.7 ± 3.0	20.7 ± 3.1	62 (37.4)	22 (13.3)	82 (49.3)
Physical activity (days/week), M ± SD	2.9 ± 1.7	3.0 ± 1.6	30 (16.3)	114 (62.0)	40 (21.7)
*Sustainability-related attitudes*					
Tap as preferred water (%)	28.8	33.2	10 (5.4)	156 (84.8)	18 (9.8)
Recycling (%)	94.6	94.6	6 (3.3)	172 (93.4)	6 (3.3)
*Screen-related recreational behaviours (hours/day)* ^c^					
Video games, M ± SD	1.6 ± 1.4	1.6 ± 1.3	57 (31.0)	75 (40.7)	52 (28.3)
Screens, M ± SD	1.7 ± 1.2	1.6 ± 1.2	42 (22.8)	78 (42.4)	64 (34.8)
Chat apps, M ± SD	1.7 ± 1.2	1.7 ± 1.2	43 (23.4)	103 (56.0)	38 (20.6)
Web online, M ± SD	1.9 ± 1.3	1.8 ± 1.2	49 (26.6)	83 (45.1)	52 (28.3)
*Addictive behaviours* ^d^					
Current smokers (%)	10.9	13.0	16 (8.7)	160 (86.9)	8 (4.4)
E-cig and HTP current users (%)	15.2	17.9	16 (8.7)	156 (84.8)	12 (6.5)
Current alcohol users (%)	37.0	41.9	37 (20.1)	127 (69.0)	20 (10.9)

BMI: body mass index; E-cig: electronic cigarette; HTP: heated tobacco product; M: mean; SD: standard deviation. ^a^ *p* value of paired *t* test < 0.05. ^b^ Categories in this case are in order of decreasing, not changing, increasing; total of 14 missing values for BMI. ^c^ For these categories, *worsening* indicates an increase in hours/day, while *improvement* indicates a decrease in hours/day. ^d^ For these categories, *worsening* indicates a change of status from non-smoker to smoker, from non-e-cigarette user to e-cigarette user, or from non-alcohol user to alcohol user, respectively; conversely, *improvement* indicates a change of status from smoker to non-smoker, from e-cigarette user to non-user, or from alcohol user to non-user.

**Table 5:** Associations between changes in the dietary score (substantially improving vs. non substantially improving and worsening vs. not worsening) according to sociodemographic and behavioural characteristics.

Characteristics	N	Substantially Improving Dietary Score (Change in Score ≥ 2) vs. Not Substantially Improving Dietary Score (Change in Score < 2)		Worsening Dietary Score (Change in Score < 0) vs. Not Worsening Dietary Score (Change in Score ≥ 0)	
		%	OR ^b^ (95% CI)	%	OR ^b^ (95% CI)
Total	184	29.4		28.3	
Sex ^a^					
Male	90	27.8	1.00	32.2	1.00
Female	92	30.4	1.20 (0.62–2.31)	25.0	0.70 (0.36–1.35)
Secondary school grade					
2nd	58	34.5	1.00	25.9	1.00
3rd	126	27.0	0.76 (0.37–1.55)	29.4	1.22 (0.58–2.58)
School type					
Technical institute	70	28.6	1.00	30.0	1.00
Professional school	28	35.7	1.22 (0.46–3.25)	28.6	1.00 (0.36–2.77)
High school	86	27.9	0.85 (0.41–1.77)	26.7	0.91 (0.44–1.86)
BMI ^a^ (tertiles; kg/m^2^)					
<19.1	59	23.7	1.00	30.5	1.00
19.1–21.4	55	25.5	1.07 (0.44–2.58)	36.4	1.32 (0.59–2.95)
≥21.4	56	41.1	**2.47 (1.07–5.71)**	17.9	0.44 (0.18–1.11)
*p* for trend			**0.034**		0.105
Change in BMI ^a^					
Reduced	62	32.3	0.60 (0.21–1.74)	32.3	2.13 (0.61–7.48)
Not changed	73	28.8	1.00	21.9	1.00
Increased	31	25.8	0.42 (0.14–1.25)	35.5	1.73 (0.48–6.18)
Physical activity frequency (days/week) ^c^					
0–1	40	32.5	1.00	27.5	1.00
2–3	81	33.3	0.97 (0.42–2.22)	22.2	0.72 (0.29–1.75)
4+	63	22.2	0.51 (0.19–1.36)	36.5	1.40 (0.54–3.60)
*p* for trend			0.158		0.368
Walking/cycling (days/week) ^c^					
0–1	68	30.9	1.00	22.1	1.00
2–3	46	28.3	0.96 (0.41–2.23)	28.3	1.37 (0.57–3.29)
4+	70	28.6	0.99 (0.47–2.09)	34.3	1.86 (0.86–4.01)
*p* for trend			0.976		0.114
Current smoking ^c^					
No	164	27.4	1.00	27.4	1.00
Yes	20	45.0	2.24 (0.86–5.87)	35.0	1.38 (0.51–3.74)
Current use of e-cig or HTP ^c^					
No	156	29.5	1.00	26.3	1.00
Yes	28	28.6	0.93 (0.38–2.32)	39.3	1.75 (0.75–4.13)
Current use of alcohol ^c^					
No	116	31.0	1.00	23.3	1.00
Yes	68	26.5	0.86 (0.44–1.71)	36.8	1.91 (0.98–3.72)

BMI: body mass index; E-cig: electronic cigarette; HTP: heated tobacco product; OR: odds ratio. ^a^ The sum does not add up to the total because of missing values: 2 missing values for sex, 14 missing values for BMI, 18 missing for BMI change. ^b^ ORs were estimated using multilevel logistic regression models after adjustment for sex, age class, and type of school, with team as random effect; estimates in bold type are significant at 0.05 level. ^c^ Variables referring to before participating in the Food Game programme (November 2024).

## Data Availability

The original contributions presented in this study are included in the article. Further inquiries can be directed to the corresponding author.
